# Thermosonication Processing of a Chickpea-Based Beverage and Its Effect on Microbiological and Physicochemical Quality

**DOI:** 10.3390/foods15132353

**Published:** 2026-07-02

**Authors:** Selene Pascual-Bustamante, María Andrea Trejo-Márquez, Juan Carlos Raya-Pérez, César Leobardo Aguirre-Mancilla, María Gabriela Vargas-Martínez, Juan Gabriel Ramírez-Pimentel

**Affiliations:** 1 Posthaverst Laboratory of Plant Products, Faculty of Higher Studies Cuautitlan, Assimilation Technology Center Jiménez Cantú s/n, San Juan Atlamica, National Autonomous University of Mexico, Cuautitlan Izcalli 54729, Mexico; spluna27@cuautitlan.unam.mx (S.P.-B.); gvargasm@unam.mx (M.G.V.-M.); 2 Tecnológico Nacional de México/IT de Roque, Carretera Celaya-Juventino Rosas km 8, Celaya 38110, Mexico; juan.rp2@roque.tecnm.mx (J.C.R.-P.); cesar.am@roque.tecnm.mx (C.L.A.-M.); juan.rp1@roque.tecnm.mx (J.G.R.-P.)

**Keywords:** legumes, non-thermal processing, plant-based beverages, *Salmonella*, food safety

## Abstract

Pasteurization is a treatment used to reduce the microbial load in beverages; however, it can affect some nutrients. The objective of this study was to evaluate the effect of using thermosonication as an alternative to pasteurization on a chickpea-based beverage. The beverage was developed with the chickpea variety ‘El Patrón’ and inoculated with a strain of *Salmonella typhi* at a concentration of 1.47 × 10 ^7^ CFU/ mL. The following treatment involved the application of ultrasound waves at different durations (15, 30, and 45 min) and amplitudes (50, 75, and 100%) at temperatures of 25 and 50 °C, while a control group was pasteurized. The effects of the treatments on the colour, protein digestibility, and viscosity of the product were evaluated. The effectiveness of the ultrasound treatment was greater when performed at 50 °C; the optimal thermosonication treatment is at 75% amplitude and 40 min. Additionally, higher viscosity was observed in the chickpea beverage treated with thermosonication (3500 mPa·s), while protein digestibility was increased by 5%. This ultrasound method is a viable alternative to thermal pasteurization in the preparation of chickpea-based beverages.

## 1. Introduction

Plant-based beverages have been part of the human diet for several centuries, with evidence of their existence from the year 20 BC. The most popular product is soy milk, for which a method was developed in the 20th century to improve its organoleptic properties and reduce its characteristic aftertaste [1].

The market for non-dairy milks or plant-based beverages in Mexico is growing. In 2021, sales of such beverages increased 13.1%, with a value of 22,259 million dollars, attributed in the population; in 2021, approximately 1500 Mexicans changed their traditional diet to a vegetarian diet, according to the international organization Million Dollar [2]. In Europe, meanwhile, Spain has led consumption of plant-based beverages, with an average of 5.20 L per person in 2020; during the COVID-19 pandemic, consumption increased by 13%, ten times than the increase recorded from 2019 [3].

To commercialize plant-based beverages, it is essential for these products to undergo a thermal process that effectively reduces any microbial load that may be present. The most widely used preservation method is pasteurization. This process in milk can be conducted under various conditions: low-temperature, extended-time pasteurization (65 °C for 30 min); high-temperature, short-time pasteurization (72 °C for 15 s); or ultra-high-temperature sterilization (136–145 °C for 2–8 s). Process conditions may vary depending on the product, and whether the process is continuous or batch-based [4]. In the case of plant-based beverages, the most commonly used pasteurization process is UHT (135 to 150 °C for short periods of 2–8 s); this process has been shown to inactivate pathogenic microorganisms; however, these conditions can damage certain components of interest, primarily heat-sensitive proteins, causing denaturation. This can lead to changes in sensory properties such as browning, a gummy texture, and bitterness, which are not particularly pleasant for consumers [5,6].

The technology that has been studied as a replacement for pasteurization is ultrasound. Its application in the food industry has been highly researched and developed, determining its effect on food properties, plant health and food processing. Technology is based on the transmission of sound through a liquid medium at a frequency above the human audible range (18 kHz) [7]. At the industrial level, some ultrasound equipment has been developed to be mainly used for the generation of emulsions, cell extraction and disruption, olive oil malaxing, wet milling and particle size reduction, homogenization and mixing, among others [8].

Ultrasound provides a method to improve traditional pasteurization or sterilization processes—through the cavitation process—which does not generate loss of nutrients in the food. The mechanism of action allows lysis of the cell wall of microorganisms, which leads to their death and, therefore, can be considered an alternative method to replace traditional thermal treatments [7]. One of the applications of ultrasound that has been studied is in the processing of milk and dairy products; however, its use in plant-based beverages made from legumes, seeds, or oilseeds has not been widely studied [9].

Some studies mention the effectiveness of ultrasound treatments in reducing microorganisms in these beverages; for example, in a study conducted on a peanut beverage in which ultrasound was applied at different intensities (200, 300, and 400 W), it was found that the treatment at 400 W achieved a logarithmic reduction of up to 0.9 [10].

Additionally, a cashew beverage treated with ultrasound was compared to a pasteurization treatment and showed a reduction in heat-labile coliforms, as well as fungi and yeasts, by 1 log CFU/mL. Concluding that no significant difference (*p* ≥ 0.05) was observed in the microbial quality of the pasteurized and ultrasonically treated product [9].

Although ultrasound is a technology that can reduce the presence of microorganisms, several studies have found that its application in combination with temperature improves the inactivation process of microorganisms, reporting positive results in the inactivation of pathogenic bacteria such as *Salmonella*, *Listeria monocytogenes* and *Escherichia coli* [11].

Some studies have reported reductions of up to 5 log CFU/mL in bacteria present in an almond beverage by applying a combination of ultrasound at 600 W and 60 °C for 40 min [12]. Another example was performed on a hazelnut beverage, where a complete inactivation of mesophilic bacteria, as well as fungi and yeasts, were found using thermosonication under conditions of 60% amplitude for 25 min and 80% amplitude for 15 min [13].

*Salmonella* is a major cause of foodborne illness. In recent years, major food poisoning outbreaks have been caused by *Salmonella* of different phenotypes [14]. Therefore, using this bacterium as a test marker to assess the effectiveness of an alternative pasteurization process is a viable approach in various studies.

In this study, it was proposed to evaluate the effectiveness of the application of thermosonication as an alternative method to pasteurization on *Salmonella typhi* in chickpea-based beverages, as well as the physical and physicochemical properties of the product, evaluating its effectiveness in inhibiting the bacteria as well as in maintaining the properties of the product.

## 2. Materials and Methods

### 2.1. Raw Materials

The plant-based beverage was made with the Desi chickpea variety ‘El Patrón’, which was harvested in 2018 from the state of Guanajuato (Mexico). The chickpeas were stored at room temperature in airtight jars until they were used.

The ingredients used were: refined sugar, Great Value commercial coconut oil and Deiman commercial flavouring. The stabilizer was provided as a sample from the Makymat company (Naucalpan de Juárez, Mexico).

### 2.2. Preparation of the Chickpea-Based Beverage

The chickpeas used for the beverage were soaked in an ultrasonic bath at 50 °C for 60 min. These conditions were based on a previous study of chickpea soaking and its effect on the removal of non-nutritional compounds [15]. After the grains had been soaked, the beverage was prepared using a formulation of 5% soaked grains, 2.5% sugar, 1% coconut oil, 1% stabilizer, and 0.5% commercial flavouring. All the ingredients were ground in an Oster food processor for 7 min; the solids were subsequently removed by passing the sample through a cloth mesh. The beverage was stored in sterilized, vacuum-sealed glass jars until it was used.

### 2.3. Effect of Ultrasonic Pasteurization on the Inhibition of Salmonella in a Chickpea-Based Beverage

To determine the effectiveness of the ultrasound treatment, challenge tests were conducted in which the chickpea-based beverage was inoculated with *Salmonella typhi* at a concentration of 1.47 × 10^7^ CFU/mL, using it as a safety indicator. The inoculated beverage was treated in a BIOBASE model UCD-500, Jinan, China, ultrasonic cell disruptor, with a frequency of 60 Hz, varying the process conditions of 15, 30, and 45 min, with amplitudes of 50, 75 and 100% applied at two temperatures, 25 and 50 °C. These results were compared with those from the pasteurization treatment with conditions of 80 °C for 15, 30 and 45 min. The pasteurization conditions were determined based on studies conducted on a hazelnut beverage and a walnut beverage [9,13].

A sample of the chickpea-based beverage was taken at a specified time (0, 15, 30 and 45 min). To achieve a more homogeneous distribution of the microorganisms, the sample was serially diluted to 10^−1^ to 10^−4^ concentrations and inoculated onto Neogen HAL001 CHROMagar (Michigan, MI, USA) plates (specific for *Salmonella*). The plates were incubated at 35 ± 2 °C for 24 h; after that time, the colonies on each plate were counted. Results were reported as CFU Log [16].

### 2.4. Optimization of the Ultrasonic Pasteurization Method as a Function of Viscosity and Colour Changes

After conducting the *Salmonella typhi* survival tests, the ultrasound treatment conditions were evaluated. To this end, a 2^2^ factorial design was proposed using Design Expert version 7.0. The chickpea-based beverage was subjected to 50 °C, varying the time (30 and 45 min) and amplitudes (75 and 100%). Response variables are viscosity which was evaluated using a Brookfield viscometer with a spindle no. 2 and a speed of 6 rpm, and product colour.

According to the statistical design, an analysis of variance was performed to determine whether there was a significant difference (*p* ≤ 0.05) in the factors (amplitude and time). Additionally, the process conditions were optimized to preserve colour and achieving lower viscosity.

### 2.5. Comparison of Pasteurization with Thermosonication on the Chickpea-Based Beverage

Upon stablishing thermosonication parameters, the effect of this treatment was compared with pasteurization, which was conducted at 80 °C for 15 min. The comparison encompassed various factors, including protein content [17], protein digestibility [18], electrophoretic profile [19], amino acid profile, viscosity, and sedimentation of the beverage. The results were analyzed using an analysis of variance (ANOVA) with a significance level 0.05 in IBM SPSS version 20.

### 2.6. Analytical Techniques

Protein. The method is based on the reaction of proteins with copper in an alkaline solution and the reduction of the Folin–Ciocalteu reagent (phosphomolybdic-phosphotungstic acid), to heteropolymolybdenum through the oxidation of aromatic amino acids, which is catalyzed by copper. The reaction is performed in an alkaline medium at pH 10 to 10.5. Bovine serum albumin was used as the standard. The tests were performed in triplicate. Results were expressed in mg protein/g sample [17].

Viscosity. Viscosity is defined as the ratio between the shear stress and the shear rate. The equipment used to determine viscosity was a Brookfield viscometer (BioBase, model BD-V8S, Jinan, China) using the L2 spindle. The tests were performed in triplicate. Results were reported in mPa·s.

Sedimentation. The sedimentation evaluation was performed by placing the beverage in a 10 mL graduated test tube and refrigerating it at 7 °C for 24 h. After this period, the separated phases were measured [9]. The tests were conducted three times for consistency and reliability.

Colour. The colour measurement of the beverage samples was performed using a KONICA MINOLTA CR600 colorimeter (Tokio, Japan). The equipment was calibrated prior to beginning the measurements, which were repeated five times, yielding the L, a, and b values. Afterward, the five readings were averaged, and calculations were performed to determine the parameters: chroma (C*) and hue (H*). The tests were performed in triplicate.

Protein profile by solubility. The Osborne method was used to perform the protein fractionation. This method consists of separating the proteins based on their solubility in the following solvents: purified water (albumins), 0.5 M NaCl solution (globulins), 70% ethanol (prolamines), and 0.1 N NAOH (glutelins). The tests were performed in triplicate [1].

Protein digestibility. This method is based on the digestion of an aqueous suspension of the test protein at 37 °C and a pH of 8, using a combination of trypsin, chymotrypsin and protease, which break the peptide bonds. The free carboxyl groups that are formed release protons lowering the pH of the protein suspension [18]. Results were reported as the digestibility percentage. The tests were performed in triplicate.

Electroforesis. This method consists of protein separation on a 10% polyacrylamide gel, using a Tris-trycine buffer solution. A vertical electrophoresis chamber was employed; gels were stained with Coomassie blue and fixed with a methanol–acetic acid mixture for 12 h [19].

Identification of amino acids in the chickpea-based beverage. Initially, the protein content of the beverage underwent hydrolysis. This involved treating the samples with a 6 N HCl solution for 24 h at 120 °C. Upon completion of the hydrolysis, the samples were neutralized with an 8 N NaOH solution to achieve a pH of 2.2. Subsequently, the hydrolyzed samples were preserved in amber glass bottles for future analyses [20].

After hydrolysis, the derivatization proceeded; in which 5 µL were taken in vials, to mix them with 30 µL of distilled water and 25 µL of OPA/2ME solution. The mixture was stirred for 1 min and allowed to stand for 2 min to continue the injection. For the separation phase, HPLC equipment (Shimadzu Brand, Model CTO-10A, Kyoto, Japan) was used where 5 µL of the derivatized sample was injected on a reverse phase AccQ●Tag^®^ column. The eluent systems used were: eluent A: 0.08 M phosphate buffer pH 7.2 and Eluent B: 0.08 M methanol-buffer phosphate pH 7.2 (55:45). The column used was Acc●QTag AccQ●Tag^®^ for amino acid analysis (3.9 mm × 150 mm), with a flow of 1 mL/min.

The amino acids were identified using a mixture of standards including the following: Alanine (Ala), Arginine (Arg), Aspartic acid (Asp), Cysteine, Glutamic acid (Glu), Glycine (Gly), Histidine (His), Isoleucine (Ile), Leucine (Leu), Lysine (Lys), Methionine (Met), Phenylalanine (Phe), Proline (Pro), Serine (Ser), Threonine (Thr), Tyrosine (Tyr) and Valine (Val). This mixture was purchased from the brand Fluka (Honeywell Fluka, Seelze, Germany) (09418), the amino acids were in a concentration of 0.01 M dispersed in a 0.1 M hydrochloric acid solution. A fluorescence detector (brand: Shimadzu, model: CBM-20, Kyoto, Japan) was used for identification.

## 3. Results and Discussion

### 3.1. Evaluation of the Effects of Ultrasound Treatments on Chickpea Beverage Inoculated with Salmonella

**Figure 1:**
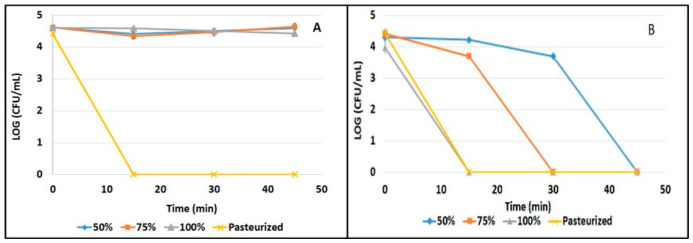
Presence of *Salmonella typhi* in chickpea-based beverage treated with ultrasound for different times and with different sound wave amplitudes at 25 °C (**A**) and 50 °C (**B**).

To determine the effectiveness of ultrasound treatments as an alternative method to pasteurization, different ultrasound process conditions were applied to the chickpea beverage inoculated with the bacterium *S. typhi*. The results are shown in Figure 1.

At a treatment temperature of 25 °C (Figure 1A), ultrasound did not sufficiently inhibit the bacteria in the chickpea-based beverage, in contrast to the pasteurized beverage, which achieved a greater inhibition of bacteria (0.6 log (Colony Forming Units: CFU)). Among the beverages treated with different ultrasound parameters at 50 °C, the abundance of *S. typhi* in the beverage treated at 50% amplitude for 30 min was 1 logarithmic unit lower than at time 0; after 45 min of treatment, the presence of bacteria in the beverage was no longer observed. At 75% amplitude, the effect on bacterial abundance was more evident, as it decreased by 2 logarithmic units during the first 15 min of treatment. After 30 min, bacterial counts were reduced to below the detection limit. The bacterial contamination of the beverages treated at 100% ultrasound amplitude was eliminated over a similar timeframe to that obtained by pasteurization: *Salmonella* was eliminated in the first 15 min of treatment, indicating greater effectiveness than that of the other proposed ultrasound treatments.

The results obtained are similar to those of other studies [21] in which various ultrasound conditions (78 and 104 W for 6 and 8 min) were tested on sheep milk, and after 4 min of treatment, coliforms initially present in the samples were eliminated. In another study performed on grapefruit juice (*Citrus maxima*) treated with ultrasound for different durations (15, 30, 45 and 60 min) and at different temperatures (30, 40 and 50 °C) [22], the microbial population (mesophiles, coliforms, fungi and yeasts) decreased as the temperature and processing time increased, and at 60 min at 50 °C, the microbial load was completely eliminated. On the other hand, it has been reported that applying ultrasound with an ultrasonic power of 100 watts achieves optimal inactivation, demonstrating effectiveness on bacteria such as *Listeria* and *Escherichia coli* [23]. In the case of an almond beverage treated with ultrasound at 60 °C, a decrease of more than 5 CFU/mL was observed in bacteria [12]. In a separate study, an ultrasound treatment was applied to a peanut beverage, resulting in a reduction of 0.9 log CFU/mL [10].

These findings are similar to those obtained in this study, where the combination of ultrasound with relatively high temperatures improved the destruction of *Salmonella* in vegetable beverage samples.

### 3.2. Optimization of the Thermosonication Method as a Function of Viscosity and Colour Changes

Once the effectiveness against *S. typhi* of the proposed ultrasound treatments was established, the conditions were optimized for use as an alternative method to pasteurization. A 2^2^-factorial design (time: 30 and 45 min; amplitude: 75 and 100%) was used to assess the combination of ultrasound and temperature (50 °C). The results obtained are presented in Table 1.

The beverages with the highest viscosity were those treated at 100% amplitude for 45 min, and the viscosity significantly differed (*p* ≤ 0.05) from that of the other treatments. The beverage with the lowest viscosity was treated by ultrasound at 75% amplitude for 30 min, with a value of 13,085 mPa·s. This change in viscosity is related to the stability of the beverage; with increasing duration and amplitude of the process, the viscosity of the beverage increased. These data are similar to those reported in the literature [24] regarding the viscosity of a fermented chickpea-based beverage to which different ultrasound treatment times (2, 4, 6 and 8 min) were applied: the viscosity was found to increase with increasing process time. This increase in viscosity may be due to the phenomenon of cavitation, which decreases the particle size because of the micro-shears generated within the suspension, which increase the number of strong interactions between particles [24]. The resulting viscosity is higher than that of cow milk, mainly because of the presence of other components, such as starch.

The luminosity of the chickpea-based beverage treated at 75% amplitude for 30 min was greatest, followed by that of the beverage treated at the same amplitude but for 45 min, whereas the luminosity of the samples treated at 100% amplitude was significantly lower (*p* ≥ 0.05). Luminosity indicates how light or dark the colour of the sample is, with 0 being the value closest to black and 100 being the value closest to white. The beverages treated at 75% amplitude were more luminous because thermosonication at this amplitude caused less oxidative effects than treatment at 100% amplitude did. This caused less browning in the beverages and therefore a difference in the luminosity of the product.

The hue (°HUE) of the chickpea beverages treated by thermosonication ranged from 2.1 to 3.1, and a significant difference (*p* ≤ 0.05) was observed between the different conditions. The hue of the beverage treated for 30 min had the highest values. The differences in tone among the chickpea beverages may be attributed to ultrasound-induced changes in the size and distribution of suspended particles. The micro shear forces generated during acoustic cavitation can disrupt particle aggregates and reduce particle size, thereby modifying light-scattering and absorption properties and ultimately affecting the perceived colour tone of the beverages. The saturation decreased as more drastic treatments were applied to the chickpea-based beverage; after the 30 min treatment at an amplitude of 100%, the lowest saturation was observed, with a significant difference (*p* ≤ 0.05) with respect to the rest of the treatments.

**Figure 2:**
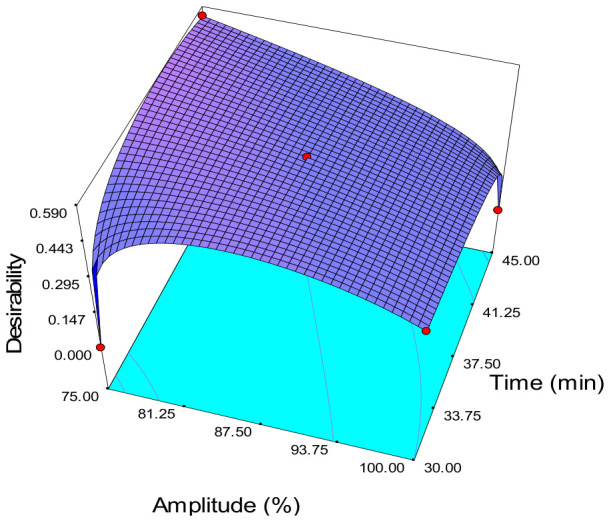
Optimization analysis according to the conditions established for the thermosonication of a chickpea-based beverage. The red dots represent the high and low levels associated with the analyzed time and amplitude conditions, as well as the midpoint for both conditions.

With respect to the results obtained for viscosity and colour, a factorial analysis was applied to predict ideal conditions for the thermosonication treatment of chickpea-based beverage (Figure 2). The results revealed that 75% amplitude for 39.76 min of thermosonication treatment were optimal for processing the chickpea beverage.

### 3.3. Comparison of Pasteurization with Thermosonication for the Chickpea-Based Beverage

The properties of beverages treated with thermosonication with the optimal conditions identified above were compared with those of unpasteurized and pasteurized beverages. Table 2 shows the results.

According to these results, there was no significant difference (*p* ≥ 0.05) in the protein content of the vegetable beverages subjected to the different treatments. This finding is similar to that of a study conducted on a cashew beverage, in which no change was detected in the macronutrients contained in the beverage before and after ultrasound treatment [9]. The protein content of the chickpea-based beverage was lower than that of a chickpea-based beverage made with coconut and reported up to 2.1 g/100 g of protein [25]. This difference may be due to the variety as well as the proportion of chickpea used in the formulation.

Among the protein fractions in the beverages, albumins are notable since they are soluble in water, reaching values of 1.1 to 1.3 g/100 g (representing almost 80% of the proteins therein) in the differently treated beverages, although the differences were not significant (*p* ≥ 0.05).

Compared with that of the pasteurized chickpea beverage, the digestibility of the beverage treated with thermosonication significantly increased by approximately 2% (*p* ≤ 0.05). In this case, none of these operations were performed; however, the ultrasound process can partially unfold the proteins contained in the chickpea beverage, which would increase the solubility of these proteins in the matrix and thus the digestibility and viscosity of the beverage. This coincides with what was found in a vegetable beverage of chickpea supplemented with linseed and treated with ultrasound: the solubility of the protein increased by 1.75% compared with that of the untreated beverage [24].

Another parameter evaluated following different treatments was the viscosity of the vegetable beverages, for which no significant difference was observed (*p* ≥ 0.05) between the chickpea beverage treated with thermosonication and the pasteurized beverage. In a previous study conducted on a chickpea-based beverage treated with ultrasound, the viscosity was greater than that of the control beverage, which may have been due to the cavitation phenomenon, which generates a greater distribution of the particles in the suspension, increasing both the stability and the viscosity of the product [24]. Viscosity is important to the sensory perception of the consumer, so it is preferable that it be similar to products that are already consumed to avoid rejection of the beverage.

In addition to viscosity and colour, sedimentation is an important quality parameter in the production of vegetable beverages. In this type of product, a stabilizer is generally applied to avoid phase separation.

No significant difference (*p* ≥ 0.05) was detected in the sedimentation of the vegetable beverage treated by thermosonication and the pasteurized beverage, with values of approximately 2%. Vegetable beverages are often characterized by sedimentation, which is due mainly to the low-molecular-weight proteins they contain. Ultrasound treatment effectively reduces particle size through the application of shear forces, turbulence, and shock waves generated by acoustic cavitation. This reduction in particle size enhances light scattering, thereby influencing key colour parameters, such as lightness (L*), and improving the visual characteristics of plant-based beverages. Additionally, a notable reduction in sedimentation was observed, which is associated with the decrease in particle size and the corresponding increase in the viscosity of the continuous phase [26]. In a study performed on a cashew vegetable beverage, ultrasound treatment improved the dispersion of the particles in the suspension and better homogenized the fat globules contained in the beverage, preventing phase separation [9].

**Figure 3:**
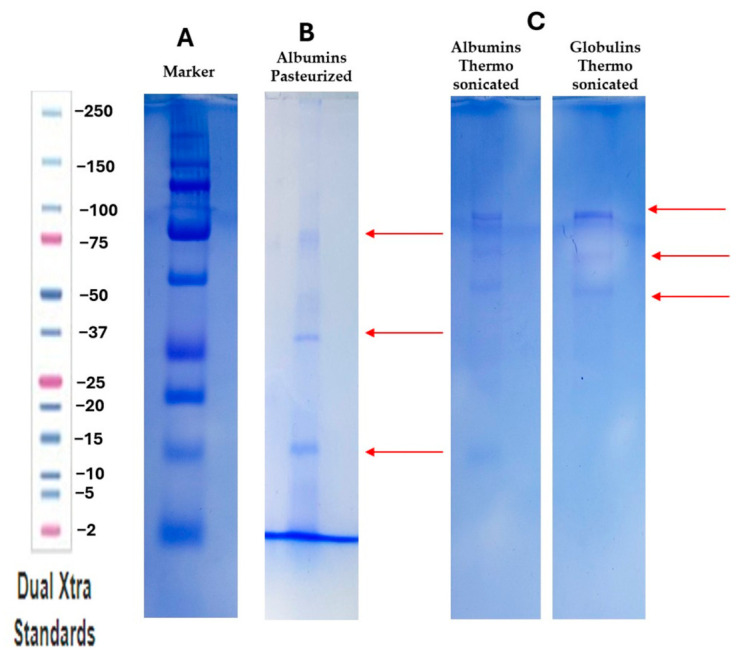
Electrophoretic profile in polyacrylamide gel of the marker (**A**) of the chickpea-based beverage treated with pasteurization (**B**) and the chickpea-based beverage treated with thermosonication (**C**).

The electrophoretic profile of the proteins in the pasteurized beverage is shown in Figure 3B, where bands are observed only in the albumin fraction, particularly those with molecular weights of 75, 50 and 20 kDa, which are characteristic of PA2 albumins.

For the beverage treated by thermosonication (Figure 3C), bands representing both the albumin and globulin fractions with weights of 100, 75 and 50 kDa each are observed in the gel. The results obtained are similar to those reported by other authors [27] for a quinoa vegetable beverage, where the marked bands reflected the albumin fraction—mainly proteins with molecular weights of 32–36 kDa. Notably, pasteurization degrades the proteins contained in the beverage, which is evident from the profile obtained; this phenomenon is not observed in the beverage treated by thermosonication, indicating that this latter process is less damaging to the protein profile. These results also coincide with those reported for lentil protein concentrates extracted by ultrasound, wherein the bands for the lentil proteins were more intense than those found in the control sample, suggesting that ultrasound facilitates the extraction and improves the preservation of proteins [28].

In terms of the amino acid profile, the pasteurized chickpea beverage presented different essential amino acids (phenylalanine, leucine, isoleucine, lysine, methionine, valine, threonine and tyrosine). Histidine was the most abundant amino acid, followed by arginine. According to the literature, chickpeas, especially the Desi variety, are rich in aspartic acid and glutamic acid, while harbouring low concentrations of tryptophan [29]. Although this test was performed on a product derived from chickpea, it is important to note the absence of tryptophan among the identified amino acids. Moreover, chickpea does contain some bitter amino acids, such as leucine, lysine, valine, phenylalanine and tyrosine, that were also present in the beverage [29].

A similar profile is observed for the chickpea beverage treated by thermosonication where histidine and arginine are accompanied by aspartic acid, which coincides with the findings in the literature regarding the abundance of these amino acids in chickpea [29]. However, it is important to note that the intensity of the signals in the beverage treated by thermosonication was approximately double that in the pasteurized beverage, which could indicate that these compounds were conserved.

The development of a chickpea-based beverage can help diversify the market for this legume and increase its consumption. It has been reported that the content of essential amino acids in chickpea flour is greater than that in wheat flour [30]. In addition, the application of thermosonication as an alternative method to pasteurization helped preserve the compounds (mainly proteins) within the product and even improved some of its characteristics.

## 4. Conclusions

According to the results of the challenge test for the inactivation of *S. typhi*, the proposed ultrasound treatments effectively reduced the detection limit for the microorganisms, specifically *Salmonella* (which was used as inoculum in the chickpea beverage). After 45 min of treatment at an ultrasound wave amplitude of 50%, the bacteria were eliminated, whereas at an amplitude of 75%, the microorganisms were eliminated after only 30 min. According to the data obtained and the results of the optimization analysis, the optimal conditions for thermosonication treatment are 75% amplitude for approximately 40 min.

Comparing the beverage treated by thermosonication with the pasteurized beverage, an increase in protein digestibility, viscosity and sedimentation was observed. The chickpea-based beverage in both treatments presented essential amino acids such as phenylalanine, leucine, isoleucine, lysine, methionine, valine, threonine and tyrosine. Thermosonication is a viable alternative to pasteurization; however, further studies are needed, emphasizing the effects on micronutrients as well as the effectiveness in reducing other microorganisms.

## Figures and Tables

**Table 1:** Evaluation of viscosity and colour of the chickpea vegetable beverage pasteurized by thermosonication.

Temperature (°C)	50			
Amplitude (%)	75		100	
Time (min)	30	45	30	45
Viscosity (mPa·s)	1308.5 ± 352.8 ^b^	2192.3 ± 474.7 ^b^	2706.45 ± 190.6 ^b^	4147.3 ± 521.4 ^a^
Luminosity	46.7 ± 0.3 ^a^	41.9 ± 0.5 ^b^	40.6 ± 0.4 ^c^	41.3 ±0.4 ^b^
Tone (°HUE)	3.1 ± 0.0 ^a^	2.1 ± 0.1 ^d^	2.6 ± 0.0 ^b^	2.2 ± 0.01 ^c^
Colour saturation	35.6 ± 1.2 ^a^	11.9 ± 1.2 ^b^	8.5 ± 0.6 ^c^	12.8 ± 0.5 ^b^

Different letters (a, b, c, d) in each column indicate a significant difference (*p* ≤ 0.05) between treatments.

**Table 2:** Effect of thermosonication and pasteurization treatment on the properties of chickpea-based beverage.

	Pasteurized Beverage	Thermosonicated Beverage
Protein (g/100 g)	1.56 ± 0.10 ^a^	1.67 ± 0.06 ^a^
Digestibility (%)	76.79 ± 0.64 ^b^	78.15 ± 0.38 ^a^
Viscosity (mPa·s)	3228.9 ± 461.2 ^a^	3491.2 ± 447.4 ^a^
Sedimentation (%)	1.78 ± 0.08 ^a^	2.06 ± 0.09 ^a^

Different letters (a, b) in each column indicate a significant difference (*p* ≤ 0.05) by treatments.

## Data Availability

The original contributions presented in this study are included in the article. Further inquiries can be directed to the corresponding author.
